# Wheat Allergy in the Era of Precision Medicine: From Novel Molecular Markers to New Therapeutic Perspectives

**DOI:** 10.3390/ijms27041717

**Published:** 2026-02-10

**Authors:** Solomiya Pukalyak, Weronika Gromek, Aleksandra Tomczak, Ewa Markut-Miotła, Maja Woźniak, Mariusz Wysokiński, Sylwia Smolinska, Emilia Majsiak

**Affiliations:** 1 Polish-Ukrainian Foundation for the Development of Medicine, Nałęczowska 14, 20-701 Lublin, Poland; solomiya.pukalyak@gmail.com (S.P.); weronikaa@gmail.com (W.G.); wozniakmaja27@gmail.com (M.W.); 2 Independent Public Healthcare Centre of the Ministry of Internal Affairs and Administration in Lublin, Grenadierów 3, 20-331 Lublin, Poland; ola.m.tomczak@gmail.com; 3 Department of Allergology and Paediatry, Medical University of Lublin, Prof. Antoniego Gębali 6, 20-093 Lublin, Poland; ewa.markut-miotla@umlub.edu.pl; 4 Department of Basic Nursing, Faculty of Health Sciences, Medical University of Lublin, Staszica 4/6, 20-081 Lublin, Poland; mariusz.wysokinski@umlub.pl; 5 Department of Clinical Immunology, Faculty of Medicine, Wroclaw Medical University, Parkowa 34, 51-616 Wroclaw, Poland; 6 Health Promotion Department, Faculty of Health Sciences, Medical University of Lublin, Staszica 4/6, 20-081 Lublin, Poland; emilia.majsiak@umlub.edu.pl

**Keywords:** wheat allergy, component-resolved diagnostics (CRDs), Tri a 37 (alpha-purothionin), Tri a 36 (LMW glutenin), omalizumab, anaphylaxis

## Abstract

Wheat allergy (WA) poses a diagnostic challenge due to its diverse clinical phenotypes—ranging from classic food allergy and wheat-dependent exercise-induced anaphylaxis (WDEIA) to baker’s asthma. An additional diagnostic aspect is serological cross-reactivity with grass pollen. Undoubtedly, the transition from extract-based diagnostics to precise component-based diagnostics (CRDs) facilitates the management of wheat allergy. It has significantly improved the diagnostic accuracy for WDEIA ω-5-gliadin (Tri a 19), although considering new knowledge about wheat proteins, it seems necessary to include them in the diagnostic scheme, especially where Tri a 19 remains negative despite clinical symptoms. Therefore, in this review, we evaluate the clinical utility of new wheat molecules with a high risk of anaphylaxis. We pay particular attention to Tri a 37 (*α*-purothionin), a thermally stable allergen associated with a 4-fold increase in the risk of severe anaphylaxis, and Tri a 36 (LMW glutenin), which shows higher sensitivity than Tri a 19 in specific pediatric cohorts. In addition, we emphasize the role of Tri a 14 (nsLTP) in distinguishing true wheat sensitization from pollen-related cross-reactivity caused by profilins (Tri a 12) or carbohydrate determinants (CCDs). Beyond diagnostics, the review discusses dynamic changes in sensitization profiles in relation to the allergic march and the phenomenon of spontaneous remission in children. New management strategies are also discussed, including the potential of omalizumab (based on the data from the OUtMATCH study) in facilitating the reintroduction of allergens into the diet.

## 1. Introduction

Wheat (*Triticum aestivum*) is a widely consumed food ingredient, complicating complete avoidance for individuals with wheat allergy or coeliac disease, necessitating an elimination diet. Furthermore, despite strict elimination, patients are sometimes exposed to wheat unknowingly, as it can be found not only in food products, but also in animal feed, cosmetics, and pharmaceutical products [1].

Wheat allergy (WA) is a complex type of food hypersensitivity, which is a reaction of the immune system to the proteins contained in wheat. WA can be a reaction dependent on immunoglobulin E (IgE) or an independent reaction. The factors that trigger the reaction are numerous wheat proteins, not only those contained in gluten, such as gliadins and glutenins, but also α-amylase/trypsin inhibitors, lipid transport proteins (LTPs), thioredoxins, and hundreds of others [1,2]. Therefore, the clinical symptoms of WA will rely on the immune mechanism, route of exposure, patient age, and specific allergenic protein. Symptoms may appear in the gastrointestinal tract, skin, respiratory system, and sometimes take a systemic form, as in the case of anaphylaxis [2].

WA is one of the food allergies significantly affecting the quality of life of patients with this disease and requiring an individual diagnostic and therapeutic approach. This review aims to comprehensively present the molecular mechanisms, current clinical challenges, and future directions in the diagnosis and management of WA, highlighting the impact of this condition on patients’ quality of life.

## 2. From Extract-Based to Component-Based Epidemiology

**Figure 1:**
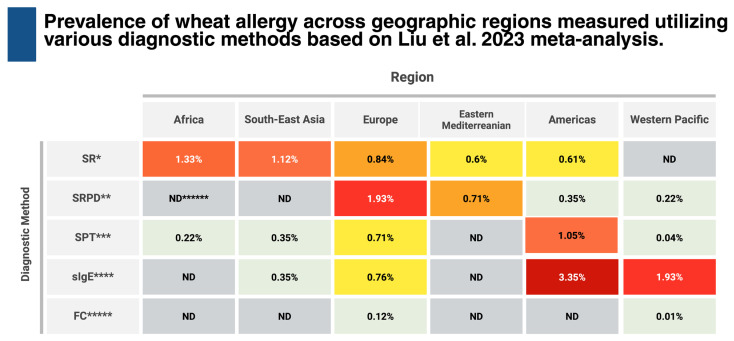
Prevalence of wheat allergy across geographic regions measured utilizing various diagnostic methods based on Liu et al. 2023 meta-analysis [3]. * SR, Self-reported; ** SRPD, Self-reported physician-diagnose; *** SPT, Skin Prick Test; **** sIgE, Specific IgE; ***** FC, Food Challenge; ****** ND, no data.

Wheat is one of the most widely consumed foods worldwide and is recognized as one of the nine major food allergens (alongside cow’s milk, eggs, soy, peanuts, tree nuts, sesame, fish, and shellfish); therefore, understanding the prevalence of WA is essential for effective public health planning and risk evaluation. However, accurate estimation of prevalence poses a challenge due to the lack of standardized diagnostic methods and a tendency to overestimate prevalence in questionnaire-based studies [3,4,5]. A meta-analysis of WA prevalence worldwide (2007–2022) revealed significant discrepancies depending on the diagnostic method used (Figure 1). The highest prevalence rates were consistently observed when utilizing sIgE measurements, peaking at 3.35% in the Americas, whereas the diagnosis based on the gold standard, the oral food challenge (OFC), yielded the lowest rates (0.01–0.12%). The diagnosis based on skin prick tests (SPT) stood in the middle of this range (0.04–1.05%).

Beyond geography, demographic and socioeconomic factors also influenced the reported prevalence (Table 1). Generally, developed countries exhibited slightly higher prevalence rates across all diagnostic methods compared to the developing nations. Interestingly, age-related trends varied by diagnostic approach: while self-reported and physician-diagnosed allergies were more frequently noted in adults, objective sensitization (sIgE) was significantly more prevalent in children (a difference of two percentage points). Furthermore, a decreasing trend in WA prevalence over time was observed across all methods, though it reached statistical significance only in the SRPD group [3].

Since there is no way to measure sIgE against individual wheat components, which may underline different clinical phenotypes of WA, we can view both the allergy itself and its prevalence from a new perspective. In wheat-dependent exercise-induced anaphylaxis (WDEIA), testing based on sIgE against wheat flour extract was effective only in 20–30% of patients with this WA phenotype. The sensitivity of detecting the cause of this reaction is significantly increased when the diagnosis is based on the determination of sIgE to one of the main wheat allergens, ω-5-gliadin (Tri a 19). Specific IgE antibodies to this allergen have been detected in 80% of the patients affected by this condition. This is because Tri a 19 is absent from water-based extracts, as this protein is soluble in alcohol [6].

Another clinical manifestation of WA is baker’s asthma (BA). It is an example of occupational asthma, induced by inhalation of wheat flour, where Tri a 14 (nsLTP) is considered as a main allergen. The estimated annual occurrence of BA is approximately 0.3%. It is the predominant type of occupational asthma in France, while in Norway and UK it ranks second in prevalence. IgE reactivity to Tri a 14 was found in approximately 60% of patients with this condition [6,7,8,9,10]. A relatively well-characterized group of wheat allergens, known as alpha-amylase inhibitors (AAI), which include Tri a 15, Tri a 28, and Tri a 29, has also been associated with BA, as well as with primary WA. IgE reactivity to Tri a 15 was observed in 60% of patients with early childhood wheat allergy (ECWA) and in 10% of those with BA. Reactivity to Tri a 28 was detected in 50–70% of individuals with WA, and reactivity to Tri a 29 was detected in 37% of individuals in the same group [6,11].

In the study conducted on a group of 3715 Polish children using component-resolved diagnostics (CRD) it was demonstrated that the sensitization rate to specific wheat molecules was 4.45% for Tri a aA_TI (α-amylase/trypsin inhibitor), 2.99% for Tri a 14 (nsLTP), and 2.35% for Tri a 19 (ω-5-gliadin). The analysis of the allergy profile depending on age showed significant dynamics of changes: the highest percentage of positive sIgE results for all analyzed wheat molecules (including markers of severe reactions) was recorded in the group of infants (<12 months of age), while in older children and adolescents (13–18 years of age), this frequency was significantly reduced [12].

New insights into the prevalence of WA may be provided by the commercial availability of sIgE testing for Tri a 36 (low-molecular-weight glutenin, LMW glutenin) and Tri a 37 (α-purothionin). In one of the first studies utilizing Tri a 36, Baar et al. [13] demonstrated that in children with IgE-mediated wheat food allergy, sIgE to Tri a 36 (57%) was identified in even more patients than sIgE to Tri a 19 (49%). Importantly, 11% of children (4 out of 37) showed sensitization to Tri a 36 while being negative for Tri a 19. Overall, the prevalence of sensitization to Tri 36 ranges from 60% to 80% among patients with WA and is approximately 5% among those with BA [6,9,13,14]. Sensitization to Tri a 36 is commonly observed in the most typical childhood phenotype of wheat food allergy [6]. Baar et al. (2012) [13] estimated that up to 0.8% of the population may be sensitized to Tri a 36. As this value does not appear explicitly in the primary studies referenced by the authors, it likely reflects an extrapolation from broader WA prevalence rather than a directly documented epidemiological figure [14,15].

In wheat food allergies, particularly in those associated with severe reactions, Tri a 37 is highly specific, although the frequency of sensitization is relatively low compared with major wheat allergens (such as Tri a 19 or Tri a 36). Tri a 37 is detected in 16–23% of individuals with WA but is absent in those with BA [9,16]. Importantly, sensitization to Tri a 37 was associated with a 4-fold increased risk of wheat-dependent anaphylaxis (OR 4.6; 95% CI, 1.5–14.2), whereas no IgE reactivity to Tri a 37 was observed in grass-pollen-allergic or non-allergic individuals [16]. 

Estimating the prevalence of WAs is not a straightforward task. The usefulness of sIgE (sensitization) to whole wheat extract is limited, mainly due to cross-reactivity with grass pollens and CCDs, which leads to clinically irrelevant, false-positive sIgE results for wheat. For this reason, CRD is recommended to distinguish true WAs (e.g., Tri a 19, Tri a 36) from clinically insignificant cross-reactivity [6,9,17,18,19].

## 3. Microbiological Classification of Wheat (*Triticum aestivum*)

Wheat belongs to the grass family Poaceae, order Poales. The most important in the context of allergology are the species of the genus Triticum, primarily *Triticum aestivum* (hexaploid; soft wheat) and *Triticum turgidum* (tetraploid; durum wheat). Common wheat (*Triticum aestivum* L.) is a hexaploid species and one of the most important crops in the world, alongside corn and rice, forming the basis of the human diet. Despite its global importance, scientists still disagree on the exact origin of common wheat (*Triticum aestivum* ssp. *aestivum*), which is associated with complex evolution through two stages of allopolyploidization and the uncertain origin of some subgenomes, such as subgenome B [20]. Research on the relationship between different species is helpful in developing the taxonomy of wheat and identifying its ancestors, but also in precisely distinguishing between species and varieties [21], which may be important from the point of view of food allergies. Taxonomic knowledge allows us to identify which species and subspecies are the source of specific allergenic proteins, which is crucial for the correct diagnosis, prevention, and effective treatment of patients with cereal allergies. In addition, it allows for the tracking of cross-reactivity between wheat and other related plants, which is particularly important for food safety and clinical practice [2]. Table 2 presents detailed classification and characterization of the most common species of wheat.

## 4. Pathomechanism of Wheat Allergy

**Figure 2:**
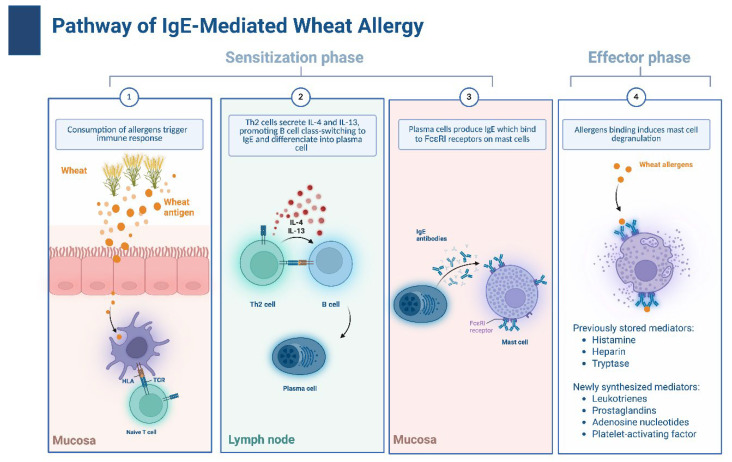
Pathway of IgE-mediated wheat allergy.

WA is a complex hypersensitivity reaction that can proceed via IgE-dependent, IgE-independent, or mixed mechanisms [1,39]. The predominant IgE-dependent reaction manifests within 2 hours of exposure. Depending on the route of entry, it is classified as food allergy (ingestion) or respiratory allergy (inhalation) [1]. The latter involves allergen contact through the respiratory tract, representing a typical route of exposure for occupational groups who have frequent contact with flour, e.g., when working in mills or bakers—so-called baker’s asthma (Figure 2).

### 4.1. The Mechanism of IgE-Dependent Reaction to Wheat

The IgE-mediated reaction is divided into two phases [40]. In the sensitization phase (phase I)*,* wheat proteins penetrate the gastrointestinal tract and the respiratory tract (mainly through inhalation of flour) or the skin (e.g., via topical exposure to cosmetics containing wheat allergens) [1,40]. After penetrating the skin or epithelial barriers, wheat proteins are captured by antigen-presenting cells (APCs), primarily dendritic cells (DCs), B lymphocytes, and macrophages, which present them as peptyides bound to major histocompatibility complex class II (MHC II). This antigen presentation leads to the activation of naïve T lymphocytes [40]. Recognition of the allergen–MHC II complex by the T cell receptor (TCR) results in T cell activation and subsequently differentiates into a T helper 2 (Th2) cells, which drive the allergic immune response. DCs play a central role in this process due to their high expression of MHC II and co-stimulatory molecules [40]. Other APCs predominantly activate memory lymphocytes [41]. When an allergen encounters the mucous membrane, epithelial cells respond by secreting alarmins, which include interleukins (ILs) such as IL-25 and IL-33, as well as thymic stromal lymphopoietin (TSLP). These activate innate type 2 lymphoid cells (ILC2). Once activated, they secrete IL-5, IL-9, and IL-13, which promotes the development of type 2 inflammation and contributes to eosinophil recruitment [40]. Type 2 inflammation is characteristic of allergic diseases, including asthma, allergic rhinitis, and food allergy [42]. After exposure to wheat allergens, allergen-specific IgE is induced. This occurs under the influence of IL-4 and IL-13, released by basophils and mast cells. These cytokines promote B lymphocytes class switching, leading to IgE production [40].

The IgE produced by B lymphocytes binds FcεRI receptors (high-affinity IgE receptors) on the surface of mast cells and basophils, sensitizing them and enabling rapid effector responses upon re-exposure to the allergen [40].

In the effector phase (phase II), re-exposure to the allergen triggers IgE cross-linking and cell degranulation. This releases pre-formed mediators (histamine, heparin, and tryptase) and newly synthesized lipid mediators (leukotrienes, prostaglandins, and platelet-activating factor (PAF)), leading to clinical symptoms [43].

This pathogenic pathway, driven by Th2 cytokines (IL-4 and IL-13) and specific IgE binding to FcεRI receptors, underpins distinct clinical phenotypes depending on the exposure route and the specific allergen involved. It manifests as a classic food allergy or wheat-dependent exercise-induced anaphylaxis (WDEIA) triggered by ingested -5-gliadin, occupational baker’s asthma induced by inhaled -amylase/trypsin inhibitors (ATIs), or contact allergy resulting from cutaneous sensitization to hydrolyzed wheat proteins (HWP) [2,6,9,11,13,16].

### 4.2. Mechanism of IgE-Independent Reaction to Wheat

An IgE-independent reaction is a reaction not associated with the production of class E antibodies but is related to immune complexes formed by food antibodies and cellular immunity. Such reactions can lead to diseases such as coeliac disease, baker’s asthma, atopic dermatitis, urticaria, and chronic gastrointestinal inflammation [44]. The reaction occurs after ingestion of wheat proteins, which penetrate the damaged intestinal epithelium, allowing allergens to reach the immune system within the intestinal mucosa. DCs capture wheat allergens and activate naive CD4+ T cells. Depending on the microenvironment, T cells differentiate into Th2, Th9, or Th17 subtypes. Th2 cells, producing IL-5 and IL-13, and Th9 cells, producing IL-9, contribute to eosinophil accumulation in the intestinal mucosa. IL-13 weakens the intestinal barrier, increasing its permeability to allergens. Accumulated eosinophils release major eosinophil protein (MBP), eosinophil peroxidase (EPO), and cytotoxic molecules, causing damage to the epithelium and symptoms such as diarrhea, vomiting, blood in the stool, and difficulty swallowing. Apart from the mechanism, the IgE-independent reaction differs from the IgE-dependent reaction in which symptoms usually appear within 2–6 h after wheat consumption. Furthermore, despite the presence of the symptoms, no positive skin test results are found and no specific IgE is detected in the blood serum [45].

Consequently, this mechanism is responsible for clinical entities such as food-protein-induced enterocolitis syndrome (FPIES) and food-protein-induced enteropathy (FPIE). It also plays a key role in mixed-type disorders, including eosinophilic esophagitis (EoE) and atopic dermatitis, where cellular inflammation coexists with antibody-mediated responses [6,9,11,13,16,17].

## 5. Wheat Proteins and Their Significance in Wheat Allergy

**Figure 3:**
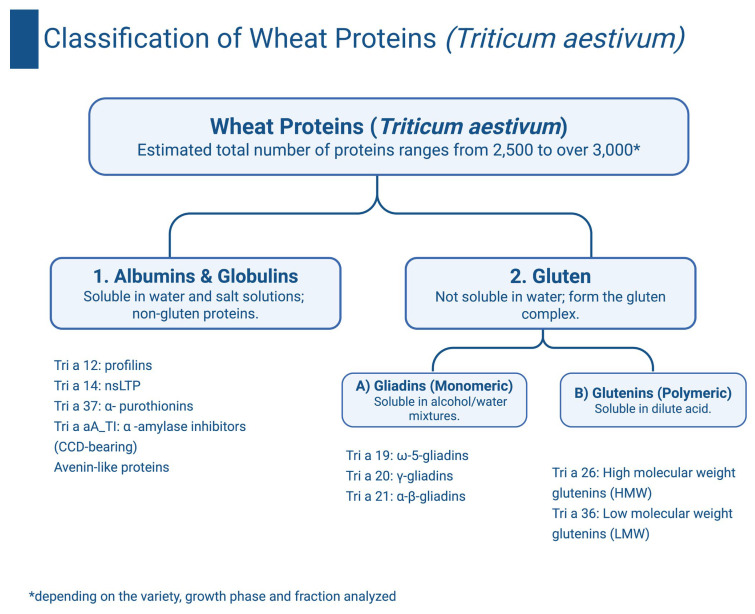
Classification of wheat proteins (*Triticum aestivum*) based on solubility properties and representative allergenic families [6,9,14,16,17].

According to the latest reports, the number of *Triticum aestivum* proteins can range from 2500 to over 3000 (depending on the variety, growth phase, and fraction analyzed) [46,47]. Wheat proteins can be divided into two main groups:

1. Water-soluble albumins and salt-soluble globulins. These include:
  - alpha-amylase inhibitors (Tri a 15, Tri a 28, Tri a 29).
  - non-specific lipid transport proteins (nsLTP)–(Tri a 14).
  - avenin-like proteins.
  - α-purothionin (Tri a 37).
2. Gluten. This is a large group of allergens, which is divided into two subgroups:
  (a) monomeric gliadins (soluble in alcohol/water):
    - α-β-gliadin (Tri a 21) and γ-gliadin (Tri a 20).
    - ω-5-gliadin (Tri a 19).
  (b) polymeric glutenins (soluble in acid):
    - high-molecular-weight glutenin (HMW) (Tri a 26).
    - low-molecular-weight glutenin (LMW) (Tri a 36) [6,9,14,16,17] (Figure 3).

Despite the large number of wheat proteins, the official list of registered and named allergens maintained by the World Health Organization (WHO) Allergen Nomenclature Committee and the International Union of Immunological Societies (IUIS) lists 28 wheat proteins as allergens. The online version of this list is available at www.allergen.org [48]. One of the most important wheat allergens is ω-5-gliadin (Tri a 19), a storage protein that plays a key role in the development of WDEIA [49]. Confirmed reactivity to wheat and a positive result for Tri a 19 correlates with the risk of severe allergic symptoms; therefore, the ability to determine sIgE to Tri a 19 increases the accuracy of WDEIA diagnosis [6,9,50].

While Tri a 19 is a key marker, another important allergen is Tri a 14, a non-specific lipid transfer protein (nsLTP), which is often the cause of allergic reactions after consumption of foods containing wheat. Belonging to the prolamin superfamily, it exhibits high thermostability and digestion resistance [49]. Due to quite high homology, sIgE to Tri a 14 causes cross-reactivity with LTPs from other plants (e.g., Tri tu 14 from durum wheat or Pru p 3 from peach). Sensitization to these proteins may be relevant in both WDEIA and isolated food reactions. Crucially, the ability to determine sIgE to Tri a 14 also allows for better differentiation between genuine wheat sensitization and pollen-related inhalant allergy, as Tri a 14 typically lacks cross-reactivity with grass pollen allergens [6,9,50,51,52].

Tri a 26 and Tri a 36 are high- and low-molecular-weight glutenins, respectively, capable of inducing IgE-mediated reactions, including severe anaphylaxis. The determination of Tri a 36 enables the identification of sensitization to gluten fractions that are poorly represented in conventional diagnostic extracts, as these proteins are water-insoluble and mainly soluble in acidic conditions. Consequently, the inclusion of these markers in molecular diagnostics enhances the detection of clinically relevant wheat sensitization beyond extract-based testing. This is particularly important in patients with a history of systemic reactions to wheat in whom Tri a 19 testing is negative [6,9,11,13,14,49].

Similarly, Tri a 37 (α-purothionin) represents a water-soluble, yet thermostable and digestion-resistant allergen, that serves as a highly specific marker for severe phenotypes. Unlike pollen-related allergens, sensitization to Tri a 37 is not associated with cross-reactivity to grass pollen or carbohydrate determinants (CCDs), making it a definitive indicator of primary wheat food allergy associated with a 4-fold increased risk of anaphylaxis. Its clinical manifestation can be modulated by gastric acidity; thus, patients using antacids or proton pump inhibitors may be at higher risk due to impaired degradation of this protein. Consequently, detecting specific IgE to Tri a 37 supports the decision to waive high-risk oral food challenges (OFC) in favor of strict avoidance of all wheat forms (including baked products) and the prescription of an adrenaline auto-injector [9,16,17].

The allergens that are fundamental in WAs are described in detail in Table 3. These include primarily Tri a 12 (profilin), Tri a 14 (nsLTP), Tri a 19 (ω-5-gliadin), Tri a 36 (low molecular weight glutenin), Tri a aA_TI (α-amylase/trypsin inhibitors), and Tri a 37 (α-purothionin) [6,11,53]. Each of these allergens is characterized by diverse structure, prevalence, resistance to physicochemical factors, and clinical significance. Their presence and detectability may be crucial for accurate diagnosis, differentiation of clinical reactions, and determination of the risk of severe symptoms such as anaphylaxis or WDEIA. Relevant details regarding IgE reactivity, solubility, stability, and diagnostic accessibility are listed in the respective rows of the table. Although Tri a 12 does not play a major clinical role, it has also been included in this characterization because its presence is important for the interpretation of a positive result obtained for wheat extract in the absence of clinical symptoms.

## 6. Clinical Picture of Wheat Allergy

WA presents with a wide range of clinical manifestations that vary depending on the underlying immunological mechanism and the route of allergen exposure. Understanding these mechanisms is essential for accurate diagnosis and appropriate management of affected individuals, as they may involve either IgE-dependent or IgE-independent pathways. These two mechanisms can also coexist, which means that symptoms can arise from this kind of mixed allergy phenotype [53].

**Figure 4:**
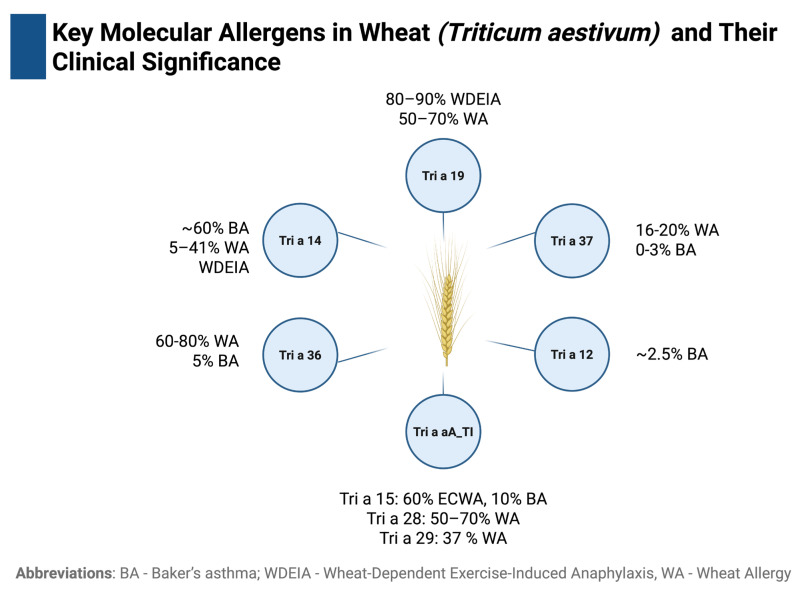
Key molecular allergens in wheat (*Triticum aestivum*) and their clinical significance.

In individuals with IgE-mediated allergies, symptoms tend to appear rapidly after contact with the allergen. Clinical signs often involve the gastrointestinal tract, leading to nausea or diarrhea. Skin reactions such as urticaria, angioedema, or other allergic eruptions are also frequently reported [3,71]. The clinical manifestations may also vary depending on the route of allergen exposure. This relationship forms the basis for distinguishing three major types of WAs: food, respiratory, and contact. Each type is associated with a distinct pattern of sensitization and symptom expression [72]. Key molecular allergens in wheat (Triticum aestivum) and their association with specific clinical phenotypes of allergy are shown in Figure 4.

Wheat is a strong food allergen that can be life-threatening, and symptoms depend on the patient’s age. In pediatric patients, symptoms related to the digestive system predominate, namely nausea, vomiting, and diarrhea; and less often—abdominal pain. Skin manifestations such as itching, erythema, urticaria, angioedema, or exacerbation of atopic dermatitis are also common. With age, gastrointestinal symptoms usually disappear, so the most common manifestation of WA in older children is skin symptoms, which may be accompanied by respiratory symptoms such as rhinitis, hoarseness, and chronic cough. These symptoms are much less common in adolescents and adults [72]. Severe reactions, including anaphylaxis caused by WA, affect different age groups, with the incidence and predominant phenotypes of these reactions varying depending on the age. The classic phenotype of anaphylaxis manifests itself as hypotensive shock, loss of consciousness, hypothermia, and cardiovascular dysfunction (tachycardia, which can progress to bradycardia) [72]. The phenotype, which depends on additional factors, is WDEIA. Typical symptoms include generalized urticaria, a drop in blood pressure, syncope, and, in children, predominantly skin and respiratory symptoms. These symptoms can occur in a person with WA after exposure to a cofactor, which may be physical activity, but not only shortly after wheat consumption [73]. The time from wheat consumption to symptom onset is 1 to 3 h, although it can be prolonged to 6 h. Physical activity is the most common trigger of WDEIA, but not the only one. Other factors that can act as cofactors for the reaction include non-steroidal anti-inflammatory drugs NSAIDs (e.g., acetylsalicylic acid (ASA), commonly known as aspirin), proton pump inhibitors (PPIs), alcohol and physical activity (of varying intensity), infections, fatigue and stress, hormonal factors (especially menstruation), fasting, fatigue, and stress [11,17,74]. There is cross-reactivity between these factors, which can lower the threshold for an anaphylactic reaction. This expanded view of cofactors has been termed “wheat allergy dependent on aggravating factors (WALDA)” [75]. These factors are crucial in the diagnosis and treatment of WA, especially in the case of WDEIA, where symptoms often only occur in combination with wheat consumption and an additional factor [11,74].

Inhalation exposure to wheat flour is a key cause of respiratory allergic diseases such as allergic rhinitis and asthma, which are recognized as occupational diseases, especially among bakers and mill workers. This type of allergy, known as baker’s asthma, is a classic example of immediate hypersensitivity, in which the inhalation of flour allergens triggers an IgE-dependent reaction. This process often begins with inflammation of the nasal mucosa, which, if left untreated or if exposure continues, can precede and lead to the development of full-blown bronchial asthma. Respiratory symptoms include sneezing, runny nose, coughing, wheezing, and shortness of breath, which usually occur within a few hours of the exposure to flour dust in the workplace [53]. In addition to respiratory manifestations, patients may experience ocular symptoms such as conjunctivitis, characterized by tearing, itching, and redness. Chronic exposure to flour can also lead to skin symptoms such as contact urticaria or dermatitis, further compromising workers’ health. The main allergens responsible for this form of allergy are water- and salt-soluble proteins such as alpha-amylase/trypsin inhibitors (e.g., Tri a 28, Tri a 29) and peroxidases, while other wheat proteins such as ω-5-gliadin are irrelevant in this context [6,73,76].

Contact allergy to wheat is a specific form of hypersensitivity that develops as a result of direct skin contact with allergens, most often in the form of hydrolyzed wheat proteins (HWP). This type of reaction is particularly common in connection with the use of cosmetics such as facial soaps, shampoos, and creams, in which wheat hydrolysate performs technological functions that increase solubility [6,53,77]. The sensitization process occurs transdermally, leading to the production of specific IgE antibodies, which distinguishes this mechanism from the classic food route. The leading clinical symptom after using the product is immediate contact urticaria, including erythema, itching, and swelling at the application site. A significant risk associated with this form of allergy is the risk of systemic reactions, including anaphylaxis, after consuming gluten-containing foods by individuals who have previously been sensitized through the skin [6,17]. In Japan, a specific subtype of WDEIA has been described, which develops precisely as a result of primary percutaneous sensitization to HWP contained in soaps, although these patients are often not sensitized to ω-5-gliadin [17]. In an occupational context, skin symptoms in the form of contact dermatitis or urticaria may also affect bakers and food service workers exposed to direct contact with flour [78]. Diagnosing this condition can be challenging, as standard tests based on native wheat extracts can produce false-negative results, so it is essential to test using wheat hydrolysate [6].

IgE-independent reactions are conditions that are not associated with circulating IgE antibodies; they are mediated by the cellular immunity. In children, WA can manifest as the food-protein-induced enterocolitis syndrome (FPIES) and food-protein-induced enteropathy (FPIE). A characteristic feature of the FPIES is a delayed onset of symptoms, which usually appear 1–4 h after the ingestion of the allergen. Children may experience persistent and recurrent vomiting and diarrhea, leading to dehydration and excessive sleepiness [79].

We can observe mixed forms of WA (IgE-dependent and IgE-independent), including eosinophilic gastrointestinal disorders (EGID), such as eosinophilic esophagitis (EoE), and dermatological diseases, such as atopic dermatitis. EoE is a mixed allergy phenotype classified as a chronic, immune/antigen-mediated disease of the esophagus. Infants may experience feeding difficulties or refusal to eat, while older children and adults may experience dysphagia, a feeling of food sticking in the esophagus, epigastric pain, and recurrent vomiting [71,79].

The clinical picture of WA can be non-specific, so a detailed medical history, examination and detailed diagnostics are necessary to make the correct and competent diagnosis and select effective treatment.

## 7. Comparison of Diagnostic Methods for Wheat Allergy

The complexity of the wheat proteome, the diversity of clinical phenotypes ranging from typical food allergy in children, through WDEIA, to occupational asthma in bakers, and the widespread phenomenon of cross-reactivity with grass pollen make the diagnosis of WA one of the greatest challenges in allergology [17]. Tests used to diagnose WA (SPT or/and sIgE) for whole wheat extract, although highly sensitive, they have significant limitations in terms of specificity [6,17]. Commercial wheat extracts used for SPT and sIgE assays contain mainly solutions dominated by albumins and globulins. It is worth noting that water-insoluble proteins are responsible for severe systemic reactions and WDEIA. Therefore, in patients with severe allergies, in whom ω-5-gliadin is the cause of the reaction, it may produce false-negative results [9,11,17]. Furthermore, studies have shown that in populations with high exposure to grass pollen, positive test results for wheat extract may occur in 60–65% of patients with grass allergy who actually tolerate wheat consumption [18,80]. The reason for this is the cross-reactivity of panallergens present in wheat, such as profilins (Tri a 12), which are homologous to grass pollen allergens (e.g., Phl p 12) and CCDs. However, this cross-reactivity, which is often seen in studies, is usually of negligible clinical significance [6,18].

In response to these limitations, CRD plays a key role in the modern diagnostic algorithm, allowing for the precise determination of the allergy profile and risk assessment [78]. The determination of sIgE for ω-5-gliadin (Tri a 19) has become a key element in the diagnosis of WDEIA and severe WA in children, showing significantly higher specificity (reaching 95–100% in some studies) compared to the extract [9,11,17,80]. Tri a 19 is a marker of severe, immediate reactions and anaphylaxis, and its concentration correlates with the risk of systemic reactions [80]. However, ω-5-gliadin alone does not allow all patients to be identified. Studies have shown that the inclusion of other gluten components in the diagnostic panel, such as HMW glutenins (Tri a 26) and LMW glutenin (Tri a 36), significantly increases diagnostic sensitivity [14]. In a study involving children with WA, the combination of sIgE analysis for Tri a 19, Tri a 26, Tri a 36, and gliadin allowed the identification of 100% of patients with a positive challenge test result, while ω-5-gliadin alone detected only 62% of cases [80].

An important element of differential diagnosis is also the assessment of sensitivity to nsLTP, i.e., Tri a 14. It is a thermally stable allergen resistant to digestion, associated with the risk of systemic reactions, WDEIA, and baker’s asthma, which does not cross-react with grass pollen [9,11]. In turn, water-soluble alpha-amylase/trypsin inhibitors (Tri a aA_TI), which are the main allergens in this group of patients, play a key role in the diagnosis of occupational asthma (bakers’ asthma), while ω-5-gliadin is usually irrelevant for them [14,81].

Clinically insignificant but diagnostically very important is the determination of markers detecting CCDs. The detection of only anti-CCDs sIgE without significant markers of WA allows for the distinction of actual sensitization from clinically insignificant cross-reactions, often resulting from the presence of homologous CCD structures in grass pollens [18,19]. We have various CCD markers (such as MUXF3 from bromelain, horseradish peroxidase, or Hom s LF), and we can determine them using various diagnostic platforms, including singleplex, multiparametric, and multiplex tests [19,61,82]. It is worth noting that, in singleplex diagnostics, testing for a specific allergen is not always automatically combined with anti-CCD detection (or requires separate detection), which gives an advantage to multiplex platforms where CCD markers are present as standard on the matrix, facilitating immediate verification of false-positive results (false in a clinical context, but diagnostically true because they reflect the actual presence of sIgE antibodies in the serum) [19]. An interesting solution used on the ALEX macroarray is the inclusion of an anti-CCD antibody blocker in the standard test protocol, which allows for automatic elimination of the interference and increased specificity of clinical results [78,83]. Multiplex tests based on statistical analysis of large data sets optimize panel compositions by removing insignificant allergens and adding missing markers, resulting in the introduction of new markers, including molecules of Tri a 36 (low-molecular-weight glutenin) and Tri a 37 (α-purothionin), which are important for the precise diagnosis of wheat allergy [62].

In vitro diagnostics are complemented by the basophil activation test (BAT), which is gaining importance as a highly specific tool, especially in cases of ambiguous sIgE and SPT results. BAT, which measures the expression of activation markers (e.g., CD63 and CD203c) on the surface of basophils after allergen stimulation, has shown high effectiveness in differentiating patients with WA from those who tolerate this cereal, as well as in identifying patients with WDEIA, where ω-5-gliadin stimulation induces strong cell activation [6,17,83].

Despite advances in serological and cellular diagnostics, the double-blind placebo-controlled oral food challenge (DBPCFC) remains the definitive reference standard (gold standard) for confirming food allergy [6]. It is particularly important in the diagnosis of WALDA, where protocols that take into account cofactors such as physical exertion, acetylsalicylic acid, or alcohol may be necessary to elicit a reaction that cannot be predicted solely based on sIgE levels [78,83].

Modern WA diagnostics have evolved from simple extract tests, which are prone to cross-reactivity with grasses, toward precise molecular diagnostics. The determination of sIgE for specific wheat molecules (Tri a 19, Tri a 14, Tri a_aATI, Tri a 26, Tri a 36, and Tri a 37) in combination with the elimination of the influence of CCDs and profilins allows for more accurate stratification of the risk of anaphylaxis, differentiation between food and inhalant allergies, and reduction in the need for risky provocation tests, which, however, remain necessary in doubtful cases.

The main diagnostic methods, together with their key characteristics and limitations, are summarized in Table 4.

## 8. Treatment and Management of Wheat Allergy

WA is one of those food allergies where the use of information obtained through CRD is particularly useful for treatment and management.

The main strategy for treating and managing WA is allergen avoidance, but increasing emphasis is placed on precision medicine to stratify patients and avoid unnecessary dietary restrictions based on individual risk assessment [84,85]. In this strategy, obtaining information about the molecular profile, where the allergy concerns Tri a 19 (ω-5-gliadin) or Tri a 14 (nsLTP), signals a high risk of severe reactions. Therefore, such a molecular profile in patients with symptoms of WA is useful for deciding whether to introduce a restrictive diet [6,9]. Furthermore, patients identified with high-risk phenotypes, particularly those with WDEIA or sensitization to storage proteins, must be equipped with an adrenaline auto-injector for self-administration as a mandatory safety measure [9,78]. When the molecular profile indicates an allergy resulting solely from cross-reactivity, for example an allergy to Tri a 12 (profilin), there is no need to introduce dietary restrictions on wheat [6,9].

In cases where the causative factor of the patient’s reaction is unclear, an OFC is performed. Here, too, CRD can be helpful, as we observe that the severity of symptoms in OFC correlates with sIgE levels for individual wheat components, but the best correlation is with the sIgE concentration for Tri a 19 (ω-5-gliadin) [80]. If the allergy profile indicates high-risk molecules, we may refrain from performing OFC, which may carry the risk of severe reactions in the patient. However, if there is a discrepancy between the clinical picture and the results of tests (serological and/or SPT) (DBPCFC), the gold standard remains essential for diagnosis [6,9,11,86]. In cases of WDEIA, provocation tests should also include the cofactor (or cofactors) [6,11,87].

As with chicken egg or cow’s milk allergy, OFC should also be considered at certain intervals after diagnosis, as a high rate of spontaneous remission of symptoms has been demonstrated in children [12,80,88]. This tendency towards tolerance acquisition is also reflected in the molecular sIgE profile. In our previous study, an analysis of the sensitization pattern by age revealed significant dynamics: the highest percentage of positive sIgE results for all analyzed wheat molecules (including markers of severe reactions) was recorded in the infant group (<12 months of age), whereas in older children and adolescents (13–18 years), this frequency decreased significantly [12]. However, specific risk factors for a prolonged course have been identified. An anaphylactic reaction before the age of 3 and high levels of specific IgE for ω-5-gliadin increased the risk of persistent WA [24]. WDEIA and baker’s asthma do not show spontaneous remission [6]. Therefore, CRD is crucial for monitoring these dynamic changes in the sensitization profile, aiding in the prediction of tolerance acquisition versus the progression towards the allergic march and persistent atopy [89].

In the management of WDEIA, it is necessary to avoid exercise within 4–6 h following wheat ingestion, as well as avoiding exercising alone or in hot or humid weather or during the pollen allergy season [11].

In the treatment of BA, the implementation of strategies to prevent exposure to flour dust through inhalation is recommended as a key element of therapy. In addition, symptomatic medication is recommended [6,9].

Currently, commercial products for specific allergen immunotherapy (ITA) are not available, but clinical trials are underway on various immunotherapy methods for IgE-mediated WA and for patients with WDEIA. A series of cases have been published in which sublingual immunotherapy (SLIT) was attempted, and in which SLIT with wheat allergen increased individual reaction thresholds (tolerance) in patients with WDEIA [6,9]. However, at present, there is insufficient published data to conclude about the appropriate product or protocols for wheat allergen ITA. CRD may be helpful in designing the safety and efficacy of ITA in WA, as it can assist in the precise selection and monitoring of future therapies, possibly based on recombinant allergens or synthetic wheat peptides.

In the era of precision medicine, therapeutic strategies for WA are evolving from strict allergen avoidance toward active pharmacological and biotechnological interventions tailored to the patient’s molecular profile. A key element of this approach is the use of omalizumab, a humanized anti-IgE monoclonal antibody. As demonstrated in the Phase III OUTMATCH study, which used a dosing protocol lasting 16 to 20 weeks, this drug significantly raises the threshold for reactions to wheat proteins, allowing 75% of patients to safely tolerate doses (600 mg of wheat protein) that would previously have caused symptoms. In terms of long-term outcomes and practical utility, data from the study indicate that after the treatment phase, 61% to 70% of participants successfully incorporated and maintained wheat in their diet (real food equivalents). Importantly, the retention rate of wheat in their diet was higher than that observed for peanuts or tree nuts (38–56%). This therapy is particularly applicable to patients with severe anaphylaxis phenotypes and multi-food allergies, serving as a “safety net” in cases of accidental exposure. Furthermore, omalizumab facilitates the introduction of oral immunotherapy (OIT) by accelerating the achievement of maintenance doses and reducing adverse events [90,91,92,93].

Parallelly, genetic engineering offers the prospect of developing hypoallergenic wheat varieties through targeted gene silencing. A prime example is the modification of ω-5-gliadin expression, which would provide a targeted solution for patients with WDEIA, where Tri a 19 is the dominant trigger. Such a personalized dietary approach would minimize the risk of systemic reactions without requiring the total elimination of wheat products in selected patient groups. Additionally, precise CRD is essential for qualifying patients for modern forms of specific immunotherapy. The use of recombinant allergens or synthetic peptides, selected based on the individual sIgE profile, may significantly enhance the safety of desensitization compared to traditional extracts [11,94,95,96].

An important aspect of WA management is education, which in WA may cover several aspects; the correct use of an adrenaline auto-injector in the first place. In patients with WALDA, additional emphasis should be placed on familiarizing the patient with possible cofactors of anaphylaxis. Patients should also be trained in the correct identification of wheat allergens on labels, what a strict wheat-free diet means, and which amounts can induce severe symptoms. Training in this area should include not only the patient and their family, but also various stakeholders, including nurseries, schools, and workplaces. At this point, it is also worth emphasizing the public awareness of food allergies. The knowledge that even trace amounts can be life-threatening for some people should be possessed by catering establishments and companies, food manufacturers, state institutions, local public authorities, and other places where patients may be unknowingly exposed to allergens that are dangerous to them. Clinical decision-making and prognostic considerations based on molecular sensitization profiles in wheat allergy are presented in Table 5. 

## 9. Summary

Wheat allergy is a condition with a diverse clinical picture that poses a serious diagnostic challenge due to the complexity of wheat proteins and the limitations of standard tests. Effective diagnosis requires the use of modern component methods and an individual approach to each patient. Following diagnosis, patients are required to adhere to a strict elimination diet and to cope with chronic stress and social exclusion, which represent indirect consequences of the condition. Therefore, to improve the quality of life of individuals with wheat allergy, it is essential to ensure not only professional medical care but also adequate psychosocial support.

People with confirmed wheat allergy and a recommended elimination diet should avoid contact with wheat, read labels carefully, check product ingredients, and remain vigilant, as in some cases the reaction can be life-threatening.

## Figures and Tables

**Table 1:** Global variability in wheat allergy prevalence depending on diagnostic method, age, and socioeconomic status (based on meta-analysis by Liu et al., 2023 [3]).

Diagnostic Method	Pooled Global Prevalence	Regional Range (Min–Max)	Age Trend	Socioeconomic Trend
sIgE (Specific IgE)	0.97%	0.35% (SE Asia)–3.35% (Americas)	Children (2.16%) > Adults (0.16%)	Developed (0.95%) > Developing (0.88%)
SPT (Skin Prick Test)	0.22%	0.04% (W. Pacific)–1.05% (Americas)	Data available mostly for children	Developed (0.28%) > Developing (0.19%)
OFC (Oral Food Challenge)	0.04%	0.01% (W. Pacific)–0.12% (Europe)	Insufficient data	Insufficient data
SRPD (Physician-Diagnosed)	0.70%	0.22% (W. Pacific)–1.93% (Europe)	Adults (1.34%) > Children (0.88%)	Developed (1.14%) > Developing (0.27%)
SR (Self-Reported)	0.63%	0.60% (E. Medit.)–1.33% (Africa)	Adults (0.83%) > Children (0.58%)	Developed (0.62%) > Developing (0.46%)

**Table 2:** Taxonomic classification of wheat with characteristics of clinically significant species [2,20,22,23,24,25,26,27,28,29,30,31,32,33,34,35,36,37,38].

Domain	Eukaryota	Organisms that have a cell nucleus and chromosomes (unlike prokaryotes) and complex organelles such as mitochondria and plastids. This domain includes a huge variety of forms: from single-celled organisms to multicellular plants, animals, and fungi. All clinically relevant cereal allergens originate from eukaryotic organisms, primarily in the plant kingdom.
Kingdom	Plantae	The plant kingdom (Plantae) includes multicellular, autotrophic eukaryotes with cellulose walls, capable of photosynthesis and unlimited growth. These organisms, which reproduce sexually or asexually, are divided into major evolutionary lines (including mosses, ferns, and seed plants), sometimes referred to in a broader sense as *Archaeplastida*. This is the most important kingdom in a clinical context, containing most of the allergenic proteins found in food and pollen. Of particular importance here are seed plants and grasses, which are the main sources of allergens.
Phylum	Spermatophyta	Plants with specialized structures (roots, stems, and conducting tissues) that reproduce by means of seeds. Seed plants are a common source of both food allergens (grains) and aeroallergens (pollen).
Subphylum/type	Angiospermae	Angiosperms (flowering plants) are a subclass of seed plants characterized by exceptional organ plasticity, which has enabled them to effectively colonize the globe. They are divided into two main classes: dicotyledons and monocotyledons, which include wheat, among others. From a clinical perspective, this group is crucial because it provides most cereal grains and strong pollen allergens. It is the species in this group that are responsible for many significant food and inhalant allergies.
Class	Monocotyledonae	Monocotyledons, also known as Liliopsida, are a class of angiosperms that include wheat and the grass family. They are characterized by the presence of a single cotyledon in the embryo, parallel leaf venation, and pollen that usually has a single pore. This group shows a high ability to adapt to harsh environments and includes key food grains: wheat, barley, rye, and corn. From a clinical perspective, this is the most important class of plants, responsible for the main food allergies to cereals and mass inhalation allergies (hay fever and asthma) caused by grass pollen.
Order	Poales	The order Poales belongs to the class of monocotyledons and includes families of key economic importance, including the most important family of grasses (Poaceae), as well as sedges and rushes. These plants are distinguished by specific characteristics, such as the presence of silica particles in their tissues and seeds with starchy endosperm. This taxon is of fundamental importance in allergology, as it groups the most important species causing allergic reactions worldwide. It is in this order that the main grass pollen allergens and food allergens of wheat, rye, barley, and oats are found.
Family	Poaceae	The Poaceae family, also known as grasses (*Gramineae*), is one of the most economically important groups of plants, comprising over 10,000 species, including wheat, corn, rice, barley, and rye. These plants are the basis of human nutrition and are characterized by enormous evolutionary success, accounting for 25% to 45% of the world’s vegetation. Plants in this family are mainly pollinated by the wind and produce large amounts of pollen. Therefore, they are one of the most important airborne allergens causing hay fever and pollen-induced asthma worldwide (“grass pollen allergy”) and include all major food allergens contained in cereals.
Genus	Triticum	The genus *Triticum* of the Poaceae family includes some of the world’s most important crops, including species with diverse genetics (di-, tetra- and hexaploid), such as common wheat, durum wheat, and spelt. The name of the genus derives from Latin (“to thresh”), and the plants belonging to it are annual or winter forms with characteristic ears. From a clinical perspective, these species, especially *Triticum aestivum*, are the primary source of protein allergens: gliadins, glutenins, and albumins. These proteins are responsible for a number of disease syndromes, including exercise-induced anaphylaxis (WDEIA), classic food allergy, and cross-reactions with other cereals.
Species	*Triticum aestivum*	Common wheat (*Triticum aestivum*) is an annual, allohexaploid species with a complex genome (AABBDD), accounting for approximately 95% of global wheat cultivation. As a domesticated form, it is the primary source of carbohydrates and protein in the human diet, containing numerous allergenic fractions (gliadins, glutenins, and albumins). Clinically, it is the main cause of food allergies, atopic dermatitis, baker’s asthma, and severe reactions, including exercise-induced anaphylaxis (WDEIA). Thermostable molecules are primarily responsible for severe systemic reactions: ω-5 gliadin (Tri a 19), LMW glutenin (Tri a 36), nsLTP (Tri a 14), and α-purotin (Tri a 37). Wheat storage proteins show high sequence homology and clinically relevant cross-reactivity with rye and barley, while reactions with grass pollen are mainly due to the presence of panallergens (profilin) and CCD.

Please note that the above division takes into account the current state of knowledge. Clinical characteristics focus on IgE-mediated reactions (food allergy, baker’s asthma, and WDEIA).

**Table 3:** List of selected wheat allergens with their characteristic features [2,6,9,11,13,14,16,48,53,54,55,56,57,58,59,60,61,62,63,64,65,66,67,68,69,70].

Allergen	Superfamily/Family/Subfamily	IgE Reactivity	Clinical Significance	Additional Information
**Tri a 12** profilin	Profilin-like/Profilin/-	~2.5% BA	Allergen smaller than wheat seeds and pollen. Cross-reactivity marker with pollen and foods, rarely clinically significant in inhalant allergy. May be one of the relevant allergens in food allergy, especially in bakeries. Rarely causes clinically significant systemic symptoms after ingestion, most reactions are mild (OAS).	Road of exposure (RoE): ingestion and inhalation. Soluble in water and salt solutions, non-glycosylated, and sensitive to heat and digestion. High sequence identity with Bet v 2 (birch pollen). Not commercially available; due to high homology within this protein family, interpretation of wheat extract results is possible based on Bet v 2 (birch profilin) or Phl p 12 (timothy grass profilin).
**Tri a 14** nsLTP	Prolamin/nsLTP/nsLTP1	~60% BA, 5–41% WA	A minor wheat allergen. The main allergen in patients with baker’s asthma. A marker of primary WA. After consumption, it may cause OAS, urticaria, and anaphylaxis. Involved in WDEIA; symptoms are often associated with cofactors (most commonly exercise, but also alcohol, infections, or drugs), which may hinder identification of the culprit. Increases sensitivity for detecting WA compared to sIgE to wheat extract No cross-reactivity with grass pollens, which aids in distinguishing WA from pollen sensitization in patients with high grass pollen sIgE. Cross-reactivity with barley nsLTP	RoE: ingestion and inhalation. Soluble in water and salt solutions, non-glycosylated, and heat/digestion resistant. Available for routine testing on singleplex (e.g., ImmunoCAP) and multiplex assay (e.g., ISAC, ALEX2, and ALEX3).
**Tri a 19** ω-5- gliadin	Prolamin/gliadin/ ω-5-gliadin	80–90% WDEIA, 50–70% WA	The major allergen in wheat seeds. Marker of primary WA. Marker of severe reactions in children with WA. The most specific marker for WDEIA (78% sensitivity and 96% specificity). Symptoms range from generalized urticaria to severe anaphylactic reactions. Allergy to this molecule is associated with persistent allergy. Cross-reacts with Sec c 20 (rye) and Hor v 20 (barley).	RoE: ingestion. Soluble in alcohol, non-glycosylated, and heat/digestion resistant. Not well represented in wheat extracts. In patients allergic to ω-5-gliadin, gluten-free diet is necessary. The best characterized single wheat allergen. Available for routine testing on singleplex (e.g., ImmunoCAP) and multiplex assay (e.g., ISAC, ALEX2, ALEX3).
**Tri a aA_TI** ATIs	Prolamin/amylase/trypsin inhibitor (ATI)/-	**Tri a 15:** 60% ECWA, 10% BA **Tri a 28**: 50–70% WA **Tri a 29**: 37% WA	Pollen and seed allergens. Major allergens in baker’s asthma. ATIs play a clear role in bakers’ eczema and asthma, as well as in food allergies. They appear to be the strongest activators of allergic respiratory responses, such as in baker’s asthma.	RoE: ingestion and inhalation. Soluble in salt solutions, glycolyzed, and heat/digestion resistant. The α-amylase/trypsin inhibitor family, which includes several 12–17 kDa proteins. May be absent in the test extract. It has been demonstrated in vitro and in animal studies that they may play a role in CD and NCGS. Available for routine testing only on multiplex assay (e.g., ISAC, ALEX2).
**Tri a 36** LMW glutenin	Prolamin/glutenin/low molecular weight glutenin (LMW glutenin)	60–80% WA, 5% BA	Major wheat food allergen. Major allergen in immediate allergy in children (common in typical childhood wheat food allergy phenotype). Major allergen in WDEIA. A potential marker for distinguishing occupational allergies from food allergies. Higher sensitivity and specificity than ω-5-gliadin for the diagnosis of wheat food allergy. Cross-reactivity with related allergens in rye, barley, oat, spelt, and rice.	RoE: ingestion. Belongs to the glutenin fraction, soluble in acids, non-glycosylated, and heat/ digestion resistant. Even after extensive in vitro gastric and duodenal digestion, Tri a 36 released distinct IgE-reactive fragments and was highly resistant to boiling. Available for routine testing only in multiplex assays (only on ALEX3).
**Tri a 37** alpha-purothionin	Thionin/alpha-purothionin	16–20% WA, 0–3% BA	Minor wheat seed allergen. Possible class I food allergen; has only linear (sequential) epitopes, a typical feature of classic type I allergens (similar to ovomucoid in egg or casein in milk). Patients with IgE to Tri a 37 have a 4-fold increased risk of severe allergic reactions upon wheat ingestion. No IgE reactivity to Tri a 37 detected in grass-pollen-allergic or non-allergic individuals Sensitization to Tri a 37 and related symptoms may be influenced by factors affecting gastric digestion, such as gastrointestinal pathologies and low stomach acidity, which can occur in children, elderly persons, or as a consequence of antacid medication.	RoE: ingestion. Belongs to albumins/globulins, soluble in water and salt solutions, non-glycosylated, and heat/digestion resistant. It belongs to the plant defense proteins and is highly expressed in wheat seeds. Available for routine testing only in multiplex assays (only on ALEX3).

**Table 4:** Comparison of diagnostic methods for IgE-mediated wheat allergy [6,9,11,12,13,14,16,17,18,19,49,78,80,83].

Diagnostic Method/Component	Target (Analyte)	Sensitivity	Specificity	Diagnostic Utility	Cost	Availability	Limitations
**SPT Wheat Extract**	Mix proteins/mainly water-soluble proteins (albumins/globulins)	Low	Low/Moderate	Basic screening	Low	Widely available	Low sensitivity (some of the gluten proteins are insoluble in water-based extracts). High false-positive rate due to grass pollen cross-reactivity.
**sIgE Wheat extract (f4)**	Mix proteins/mainly water-soluble proteins (albumins/globulins)	Low/moderate	Low	Basic screening	Low	Widely available	Low sensitivity (some of the gluten proteins are insoluble in water-based extracts). Low specificity (cross-reactivity with Phl p 12/profilins and CCDs). Poor predictor of clinical reactivity without CRD.
**sIgE Tri a 19**	ω-5 gliadin	Moderate (50–70% WA)/ high (~80% in WDEIA)	High (95–100%)	Second-line diagnostics	Low	Widely available	May miss ~20–30% of WDEIA cases. Often negative in children with immediate WA (who react only to Tri a 36); cofactor-dependent clinical expression, e.g., exercise and NSAIDs).
**sIgE Tri a 36**	LMW glutenin	High	High	Second-line diagnostics	Moderate (multiplex-based)	Available, but limited to one diagnostic platform	Available mainly in multiplex assays (only on ALEX3).
**sIgE Tri a 37**	α-purothionin	Low	High	Second-line diagnostics	Moderate (multiplex-based)	Available, but limited to one diagnostic platform	Low sensitivity (detected in minority of patients) but indicates 4-fold increased risk when positive. Available mainly in multiplex assays (only on ALEX3).
**sIgE Tri a 14**	non-specific lipid transfer protein (nsLTP)	Moderate	High	Second-line diagnostics	Low	Widely available	Possible cross-reactivity with other plant nsLTPs and cofactor-dependent clinical expression (e.g., exercise and NSAIDs).
**sIgE Tri a 12**	Profilin	Low	Low	Second-line diagnostics	Non-routine testing	Limited; not routinely available	Marker of cross-reactivity with grass pollen (e.g., Phl p 12). Indicates low risk of systemic reaction.
**Multiplex (e.g., ALEX)**	Multiple components + CCD marker	High	High	Comprehensive molecular profiling First-/second- diagnostics line (top-down /bottom-up approaches)	Moderate (per test); low per analyte	Routine availability with regional variability	Component availability depends on the test version; higher cost and limited accessibility compared with standard serology; ongoing need for large-scale validation.
**BAT (Basophil Activation Test)**	Basophil surface markers (CD63/CD203c)	High	High	Functional second-line test	High	Limited availability/specialized laboratories only	Technically demanding; requires fresh blood, specialized equipment (flow cytometer), and validation. ~10% of population are non-responders.
**BBEA (Bead-Based Epitope Assay)**	peptide epitopes (*n* = 79) (key: ω-5 and γ-gliadin sequences)	High	High	Research tool with high diagnostic potential	High	Research use only for wheat/low availability for penut	High cost; currently not commercially available for wheat in routine practice.
**OFC (Oral Food Challenge)**	Clinical reaction	Gold standard	Gold standard	Reference diagnostic standard	High	Limited; hospital-based procedure	Risk of severe anaphylaxis; resource-intensive; requires precise cofactor protocols.

**Table 5:** Clinical decision-making and prognosis based on molecular sensitization profiles in wheat allergy [6,9,11,12,16,17,78,80,84,85,86,87,88,89,94].

Molecule	Clinical Phenotype/Anaphylaxis Risk	Management Strategy (Clinical Decision)	Monitoring and Prognosis
**Tri a 19** **(ω-5-gliadyna)**	Wheat-dependent exercise-induced anaphylaxis (WDEIA)/severe IgE-mediated allergy.	Strict gluten-free diet (for resting anaphylaxis) or, in WDEIA, avoidance of gluten combined with cofactors (exercise, NSAIDs, and alcohol). Adrenaline auto-injector is recommended. OFC (oral food challenge): may be waived if risk is high; WDEIA challenges must include cofactor(s). For WDEIA, avoid exercise (including brisk walking) for 4 h after ingestion.	Risk of persistent allergy: high sIgE to Tri a 19 is a marker of persistent allergy. WDEIA and baker’s asthma typically persist for life. Education on cofactors is essential.
**Tri a 37** **(α-purotionina)**	High risk* of severe anaphylaxis (cofactor-independent). *a 4-fold increased risk of severe reactions.	Strict gluten-free diet. Adrenaline auto-injector is mandatory. Caution with OFC qualification—may be waived due to severe risk. Diet should often exclude not just wheat, but also rye and barley due to >80% sequence identity.	Guarded prognosis. High sIgE levels are associated with slower tolerance acquisition. Clinical manifestation may be modulated by gastric acidity; patients on antacids/PPIs may be at higher risk due to impaired degradation.
**Tri a 14** **(nsLTP)**	Cross-reactive or cofactor-dependent allergy. Baker’s asthma (major allergen). Risk of systemic reactions (anaphylaxis, urticaria).	Wheat elimination often necessary. No cross-reactivity with grass pollen–aids in differential diagnosis from pollen allergy. OFC: May be waived if risk is high; in WDEIA cases, challenge must include cofactors. For Baker’s asthma: reduce occupational exposure (masks, job change).	Individual monitoring. Assess tolerance based on cofactors. Potential cross-reactivity with other LTP-containing plants (e.g., peach, barley). Patient education regarding cofactors is crucial.
**Tri a 36** **(LMW glutenina)**	Typical wheat food allergy in children. WDEIA Major allergen in immediate reactions in children (higher sensitivity than Tri a 19 in this group).	Wheat elimination diet. An adrenaline auto-injector is mandatory in patients at risk of anaphylaxis. In case of discrepancy between history and serology, confirmation via OFC is indicated. Differentiates true food allergy from grass pollen cross-reactivity.	High probability of acquiring tolerance. Regular monitoring of sIgE is recommended-annual OFC in early childhood is advised to assess the development of tolerance.
**Tri a 12** **(Profilin)**	Mostly asymptomatic sensitization or mild oral allergy syndrome (OAS). Marker of cross-reactivity with grass pollen (e.g., Phl p 12).	No indication for elimination diet (unless clinical symptoms are confirmed). Positive result usually reflects cross-reactivity; OFC only if actual clinical symptoms occur. Sensitization to profilins is usually secondary to pollen allergy; detection of Tri a 12 in asymptomatic patients does not justify an elimination diet and should prompt evaluation for pollen (especially grass) allergy.	Not applicable. Symptoms are typically seasonal and related to grass pollination periods.

## Data Availability

Data sharing is not applicable to this article as no new data were created or analyzed in this study.
